# Editorial

**Published:** 1856-09

**Authors:** 


### THE MEDICAL EXAMINER.

### PHILADELPHIA, SEPTEMBER, 1856.

New York and Philadelphia.—An article with the above caption appears in the July number of the American Medical Monthly, of New York, under the head of editorial and miscellaneous, which, for the credit of said Journal, we rather prefer to consider miscellaneous than editorial. It smacks altogether too much of provincialism to be the performance of a genuine Knickerbocker, and we shall require better authority than its own intrinsic merits to convince us that it is the production of any one entitled to represent the local "feelings" and interests which it would lead us to regard as so cruelly outraged. We do not consider ourselves, however, as at all called upon to defend Dr. Neill, the Publication Committee, our Medical Schools, Grinders, Publishers, and we can't remember how many more, who are so unfortunate as to be the subjects of this absurd attack. We may remark, however, *en passant*, that the unworthy attempt to create the impression that the disturbance which so unexpectedly grew out of the resolution offered at Detroit arose from a feeling of jealousy against New York, is so preposterous, that we can hardly think it will impose upon any one,—particularly when it is known that Dr. N. had not consulted with a single one of his colleagues before offering his resolution, although he *had* conferred with some of the most distinguished members of the New York delegation, who fully concurred with him in the propriety of the movement. Our object, however, in noticing the article, was not to defend Philadelphia against the aspersions made upon her, nor the resolution, which speaks for itself, but to ask the attention of our readers to a few remarks upon the following extract. It reads thus:—

"If New York demonstrates to the satisfaction of the stupidest, that it is possible to publish the Transactions better, and more economically by between five and ten hundred dollars a year, Philadelphia clutches back the right to publish, though it does not dare to ignore the lesson."

Any intelligent reader might have predicted that no verification would accompany the above assertion. Had the writer restricted himself to the simple statement that printing is done more cheaply in New York than in Philadelphia, we should have contented ourselves with the remark that the reverse is the truth, and left the demonstration of the fact to those who should care to enter upon it. Had he affirmed that New York does better printing than Philadelphia, we should have submitted the 7th and 8th volumes of the Transactions of the American Medical Association to our readers, and confidently abided the judgment of the profession. But when we are quietly assured that our National Association would have saved "five or ten hundred dollars" by printing its Transactions in New York instead of Philadelphia, simple affirmation should give place to demonstration. This demonstration we are happily enabled to furnish, in such a way that our readers must be careless indeed should they fail to understand it.

Let the reader compare the 7th and 8th volumes of the Transactions of our National Association. We do not think it is partiality which induces us to assign a preference for the latter, so far as respects the paper on which it is printed, or the beauty and accuracy of its mechanical execution.

| | |
|---|---|
| The bill rendered for printing the 7th Vol. was - - - - | $1372 61 |
| do. do. for 8th Vol. - - - - - - | 1620 79 |
| Difference | 248 18 |

Here is an apparent difference against Philadelphia. But, if the reader will carefully follow us, he will see that this difference will eventually appear against New York. The degree in which tabular matter exceeds the cost of plain matter is well known to all who have the slightest familiarity with the press. Now, let the reader cast his eye over the pages of Vol. 8, and he will at once notice an unusually heavy quantity of tabular matter—121 pages; while in Vol. 7 there are but 15 pages of such matter. Now, tabular matter (rule and figure work) not only costs double that of plain matter, but the cost of such matter is additionally enhanced by the fact that it is usually set in smaller type than the text of a given work. Let us assume that an ordinary page of the Transactions costs 75 cents, now, a page of brevier rule and figure work will cost $2 50. The number of tabular pages in Vol. 8 exceeds those in Vol. 7 by 106, which, at $1 75 per page, would give us $185 50, reducing thus, by that amount, the difference between New York and Philadelphia bill. Any reader can at once appreciate such distinctions as these. But we rely on very different data to make the question in dispute intelligible to him.

| | |
|---|---|
| The cost of setting the type for Vol. 7, - - - - - - | $514 41 |
| do. do. do. 8, is - - - - - | 822 ~~51~~ 15 |
| Difference | ~~308 10~~ 307 74 |

Thus, it cost *three hundred and seven dollars and seventy-four cents* more to set the type for Vol. 8 than for Vol. 7. Deduct from this amount the difference already alluded to, of New York bill against Philadelphia bill, and we have a difference against New York of $59 56. But this is not all. *Eleven hundred* copies of Vol. 8 were printed; while of Vol. 7 there were but 1000 copies printed. 100 extra copies on 48 forms will require $5\frac{1}{3}$ reams of paper, which, at nine dollars per ream, cost forty-eight dollars, increasing by that amount the difference against the New York bill. Not only this; but Vol. 8 exceeds Vol. 7 by *ninety-six* pages, to print 1000 copies of which, required $6\frac{2}{3}$ reams, which, at nine dollars, augments the difference against New York by sixty dollars more. If we add to these items 48 half tokens of press-work on 48 forms, and 24 tokens on ninety-six pages, in all, 48 tokens a 40 cts., we shall find the difference against New York still further increased by $19 20; total balance against New York, *one hundred and eighty-seven dollars and thirty-six cents.* ($187 36.)

As regards the first six volumes issued in Philadelphia, the cost of printing, we may observe, was considerably less than that of Vol. 8.

We have thus shown that Philadelphia printed the *Transactions* 10 per cent. more cheaply than New York. How much better, the profession must determine.

